# Transmission Interruption of Leprosy in the Philippines: An Update on the Current Program Priorities and Interventions

**DOI:** 10.3390/tropicalmed11070192

**Published:** 2026-07-09

**Authors:** Bayo Segun Fatunmbi, Alexander Yabes Taruc, Kazim Hizbullah Sanikullah, Anna Marie Celina Garfin, Jose Gerard Belimac, Almira Cruz Gatchalian, Ma. Regina De Jesus Valdez, Carmel Angela Buado, Kim Patrick Tejano, Abelaine Venida-Tablizo, Frederica Veronica Marquez-Protacio, Belen L. Dofitas, Arturo Cunanan, Reginald Alain R. Santos, Francesca Cando Gajete, Concepcion P. Dumawat, Eugene Caccam, Eunyoung Ko, Rui Paulo de Jesus

**Affiliations:** 1 World Health Organization—Philippine Country Office, Manila City 1003, Philippines; koe@who.int (E.K.); jesusr@who.int (R.P.d.J.); 2 World Health Organization—Western Pacific Regional Office, Manila City 1000, Philippines; sanikullahh@who.int; 3 Department of Health, Manila City 1003, Philippines; aggarfin@doh.gov.ph (A.M.C.G.); jbbelimac@doh.gov.ph (J.G.B.); acgatchalian@doh.gov.ph (A.C.G.); mrjvaldez@doh.gov.ph (M.R.D.J.V.); cpbuado@doh.gov.ph (C.A.B.); kim.tejano@who.int (K.P.T.); 4 Leprosy Interest Group, Philippine Dermatological Society, Quezon City 1001, Philippines; abbyvenida@yahoo.com (A.V.-T.); via_moo@yahoo.com (F.V.M.-P.); bldofitas@up.edu.ph (B.L.D.); 5 Culion Sanitarium and General Hospital Palawan, Palawan 5315, Philippines; artculsan@gmail.com; 6 Philippine Leprosy Mission, Quezon City 1003, Philippines; greentravelerph@gmail.com; 7 International Leprosy Association, Greenville, SC 29601, USA; francesca_gajete@yahoo.com; 8 Culion Foundation Inc., Quezon City 1103, Philippines; cdumawat@yahoo.com (C.P.D.); eugenecaccam@gmail.com (E.C.)

**Keywords:** leprosy, transmission interruption, Philippines, World Health Organization, neglected tropical diseases, public health surveillance

## Abstract

The Philippines achieved World Health Organization (WHO) certification for the elimination of leprosy as a public health problem in 1998. Despite this milestone, new cases continue to be reported each year, highlighting the need for sustained surveillance and interventions to achieve zero transmission. This paper provides an update on the country’s progress toward interruption of leprosy transmission using national surveillance data and programmatic reports from 2020–2024. Quantitative data were obtained from the Department of Health (DOH) Field Health Services Information System (FHSIS), while policy and programmatic information were drawn from national reports, WHO guidance, and implementation reviews. Descriptive analyses were conducted to examine trends in prevalence, case detection rates (CDRs), and age-sex distribution patterns to identify high-risk groups. Leprosy prevalence declined from 0.41 per 10,000 population in 2020 to 0.11 in 2024, remaining below the WHO elimination threshold. The CDR increased from 0.45 in 2022 to 1.17 per 100,000 in 2024, indicating recovery of active surveillance after COVID-19-related disruptions. Most newly detected cases occurred among adults aged 20–59 years (72%), although continued detection among children aged 0–14 years (6–7%) suggests ongoing transmission in selected endemic areas. Key program strengths include policy integration and WHO-supported surveillance initiatives, while major barriers include stigma, uneven local implementation, and limited access to rehabilitation. The Philippines has maintained low national prevalence while strengthening efforts toward transmission interruption. Continued investment in surveillance, contact tracing, stigma reduction, and integrated neglected tropical disease (NTD) services will be essential to achieving zero transmission, zero disability, and zero discrimination by 2030.

## 1. Introduction

Leprosy (Hansen’s disease) is a chronic infectious disease caused by *Mycobacterium leprae* and *Mycobacterium lepromatosis* that primarily affects the skin and peripheral nerves. More than 200,000 cases are still reported each year globally, with the highest burden in Asia and Africa. Although the Philippines achieved World Health Organization (WHO) certification for the elimination of leprosy as a public health problem in 1998 [1], the country continues to detect between 1000 and 10,000 new cases annually [2]. These persistent cases highlight the challenge of interrupting residual transmission in localized endemic areas.

In line with the WHO Global Leprosy Strategy 2021–2030 and the Sustainable Development Goals (SDGs), the Department of Health (DOH) aims to interrupt local transmission by 2030 [3] Current strategies emphasize integrated surveillance, active case detection, chemoprophylaxis, disability prevention, and stigma reduction within the broader neglected tropical disease (NTD) and universal health care (UHC) frameworks.

The Philippines has a long history of leprosy control dating back to the early 1900s [4] Early responses relied heavily on isolation and segregation policies (1907–1952). The Philippine Commission passed the Republic Act 1711 in 1907 [5], allowing the Director of Health to apprehend, detain, isolate, segregate, or confine individuals with leprosy. Patients were forcibly taken from their homes and brought to Culion, a former leper colony in Palawan, to live in isolation. This approach was based on the belief that leprosy was highly contagious and incurable.

In 1952, emphasis shifted towards diagnosis and treatment. The Republic Act No. 753 [6], Amended Act 1711, introduced medical inspection and diagnostic procedures to confirm leprosy cases. And individuals suspected of having leprosy were subjected to medical inspection, and only those positively diagnosed were isolated and segregated. From 1964 onwards, advances in Medical Science and Treatment (1964) [7] have shown the discovery of effective treatments. Republic Act No. 4073 [8] further liberalized leprosy treatment, allowing patients to receive treatment in government skin clinics, rural health units, or from licensed physicians. It institutionalized the treatment, which was only required for patients with advanced stages of the disease.

In January 2024, the Philippine Department of Health launched an initiative—the Multi-diseases Elimination Plan (MDEP) [9] The initiative identified 13 diseases, including leprosy, earmarked for elimination by 2030 [10]. It aimed at integrated approaches to key pillars of surveillance and information systems, access to laboratory services, service delivery, safe and quality medicines, vaccines and technology, human resource and capacity building, environment and social determinants of health, stewardship and finance, and research. It envisioned the Philippines with zero or a significantly reduced number of new infections of priority diseases, such as leprosy, for elimination through an effective health care system by 2030.

### 1.1. Review of Evidence on Leprosy Transmission Interruption

Global and regional evidence has increasingly shaped the technical approach to leprosy elimination. Achieving zero transmission requires moving beyond passive case detection and MDT delivery toward a more proactive, contact-centered strategy. This section reviews key evidence underpinning the interventions central to the Philippines elimination pathway.

#### 1.1.1. Post-Exposure Prophylaxis (PEP) and Chemoprophylaxis

The most significant advance in transmission interruption has been the evidence base for single-dose rifampicin as post-exposure prophylaxis (SDR-PEP). The landmark COLEP cluster-randomized controlled trial in Bangladesh, the largest and most methodologically robust trial of its kind, demonstrated that a single dose of rifampicin given to close contacts of newly diagnosed patients reduced the incidence of leprosy by 57% (95% confidence interval 33% to 72%) at two years [11] The subsequent international Leprosy Post-Exposure Prophylaxis (LPEP) programme demonstrated the feasibility of integrating contact tracing and SDR-PEP into routine national leprosy programmes across seven endemic countries, with moderate-quality evidence and longer-term follow-up in highly endemic settings suggesting effectiveness of 35–40% at ten years [12].

More recently, the PEOPLE trial [13] evaluated a higher double-dose rifampicin regimen compared to standard SDR-PEP across communities in Madagascar and Comoros, representing the most current effort to optimize prophylactic dosing for high-endemicity settings. The World Health Organization (WHO) recommends contact screening accompanied by SDR administration to eligible contacts as a core component of leprosy prevention [14]. In the Philippines, the Department of Health (DOH) Administrative Order No. 2021-0004 formally institutionalized SDR-PEP as part of the national leprosy control strategy, mandating chemoprophylaxis with a single dose of rifampicin for all household contacts of index cases aged two years and above, after excluding active leprosy and tuberculosis and in the absence of other contraindications, with implementation contingent on adequate contact management and consent of the index patient [15].

#### 1.1.2. Active Case-Finding and Contact Tracing

Evidence consistently shows that household contacts of leprosy patients face substantially elevated infection risk, estimated at approximately nine times higher than unexposed households, with neighbors and social contacts also at risk [16] Expert consensus has established that a successful transmission interruption programme requires early diagnosis and prompt multi-drug therapy (MDT) for all patients, systematic contact tracing and PEP, improved diagnostic tools, and rigorous epidemiological surveillance [17]. Studies in the Philippines underscore this urgency: population-based molecular epidemiology using Variable Number Tandem Repeat (VNTR) and Single Nucleotide Polymorphism (SNP) typing in Cebu found that *M. leprae* transmission is ongoing among the population under 40 years of age in both urban and rural settings, with multicase families indicative of localized transmission from shared sources [18]. This was corroborated by an eleven-year retrospective epidemiological study in Cebu (2000–2010), which found that despite high MDT coverage and Bacillus Calmette-Guérin (BCG) vaccination rates in children, leprosy case notification rates did not decline as expected following national elimination, pointing to uninterrupted local transmission [19].

### 1.2. Implementation Framework for Zero Transmission of Leprosy in the Philippines

The Philippine Pathway to Zero Transmission is aligned with the WHO Global Leprosy Strategy 2021–2030, which aims to:

1. Stop leprosy and its complications.
2. Stop discrimination and promote inclusion.
3. Strengthen government ownership and partnerships.

This framework reflects the Philippines’ commitment to achieving zero transmission, zero disability, and zero discrimination. The Philippines has adopted a comprehensive and integrated pathway toward zero transmission of leprosy, fully aligned with the WHO Global Leprosy Strategy 2021–2030 [14]. This implementation framework builds three interrelated strategic directions that reflect the country’s commitment to stop transmission, end disability, and eliminate discrimination associated with leprosy.

First, stopping leprosy and its complications focuses on strengthening early detection, ensuring prompt treatment, and preventing disability. The program prioritizes the scaling up of active case detection through integrated skin NTD surveillance, contact tracing, and screening in high-risk areas. Universal access to WHO-recommended Multi-Drug Therapy (MDT) is maintained, alongside the implementation and expansion of chemoprophylaxis such as single-dose rifampicin for eligible contacts. Efforts also emphasize enhanced morbidity management and disability prevention through the integration of physiotherapy, self-care, and rehabilitation services within primary health care, complemented by post-treatment surveillance to monitor relapse and residual disabilities.

Second, stopping discrimination and promoting inclusion highlight the importance of empowering persons affected by leprosy and addressing stigma at all levels of society. The program integrates stigma reduction strategies into national and regional health promotion activities and ensures the active participation of affected persons in advocacy, policy development, and community education. Preserving historical narratives and personal stories is recognized as a key element in promoting empathy, combating misconceptions, and fostering social acceptance. Communication campaigns are designed to be rights-based and culturally sensitive, emphasizing that leprosy is curable, and that early detection prevents disability.

Finally, strengthening government ownership and partnerships ensures program sustainability and multisectoral collaboration within the Universal Health Care (UHC) framework. Domestic financing and local resource mobilization are being reinforced to sustain surveillance, treatment, and rehabilitation services. Future integration of leprosy and other skin NTD services into primary and community health systems will enhance efficiency and accessibility. Coordination among health, social welfare, education, and local governance sectors is vital to address the broader determinants of leprosy. Strong partnerships with WHO, NGOs, academic institutions, and communities foster innovation, operational research, and capacity building, while digitalized monitoring systems and regular program reviews strengthen accountability and performance-based planning.

Through this framework, the Philippines aims to achieve and sustain zero leprosy transmission, ensure no one suffers disability due to delayed care, and guarantee dignity, inclusion, and human rights for all persons affected by leprosy.

WHO offers technical guidance on the interruption of transmission and the elimination of leprosy.

**Figure 1:**
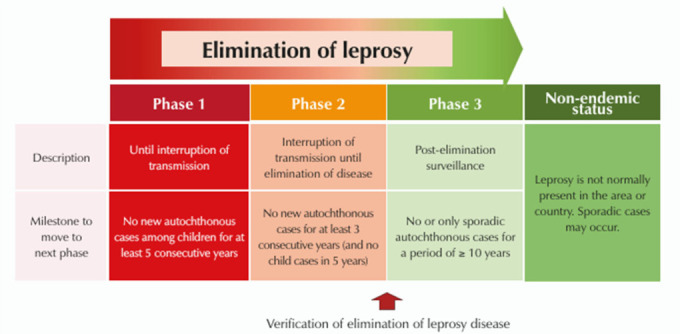
WHO framework for the elimination of leprosy: three phases.

The WHO Interruption of Transmission and Elimination of Leprosy disease Technical Guidance provides a comprehensive framework [20] as seen in Figure 1, guiding countries toward the elimination of leprosy as a disease and the ultimate interruption of transmission. This framework envisions elimination as a progressive, phased process encompassing three core phases: (1) Reduction in disease burden, achieved through early case detection, universal access to multidrug therapy (MDT), and prevention of disabilities; (2) Interruption of transmission, characterized by the absence of new child cases and zero Grade-2 disabilities among newly detected individuals, supported by expanded chemoprophylaxis, strengthened surveillance, and integrated skin NTD approaches; and (3) Verification and post-elimination surveillance, ensuring that transmission remains interrupted through continuous monitoring, periodic assessments, and integration of leprosy surveillance within the broader health system.

For the Philippines, this WHO framework provides the strategic foundation for the national and subnational pathway to zero transmission. The country has already sustained the elimination of leprosy as a public health problem since 1998, maintaining prevalence below 1 per 10,000 population. Current efforts now focus on consolidating progress toward the second phase (interruption of transmission) through intensified case-finding, early diagnosis, and the implementation of chemoprophylaxis among contacts. As endemic pockets become more localized, emphasis is placed on post-exposure prophylaxis, integrated skin NTD surveillance, and community awareness to detect and treat remaining cases promptly. Transitioning to the third phase will require a robust post-elimination surveillance system that is country-owned, data-driven, and set within the Universal Health Care (UHC) platform.

Aligned with the WHO Global Leprosy Strategy 2021–2030, the Philippine approach emphasizes “zero transmission, zero disability, and zero discrimination.” Through adherence to WHO technical guidance and strengthened national implementation, the country aims not only to sustain elimination but to achieve full interruption of leprosy transmission and safeguard future generations from the disease.

To support countries in monitoring progress along the elimination pathway, the Leprosy Elimination Monitoring Tool (LEMT) [21], with an Excel-based instrument developed to track key indicators across the different phases of elimination and to determine eligibility for progression from one phase to the next. Complementing this, the Leprosy Programme and Transmission Assessment (LPTA) [22] serves as a comprehensive evaluation framework that assesses both programme performance and transmission status against a defined set of criteria covering all critical aspects of service delivery. The LPTA may be conducted at the subnational level prior to formal acknowledgement by the Ministry of Health that a particular area has achieved interruption of transmission or elimination, but it can also be applied periodically to guide continuous quality improvement. Importantly, WHO will utilize the LPTA at the national level as part of the verification process to confirm that a country has met and sustained the milestone of leprosy elimination.

### 1.3. Collaboration and Partnerships

Strong collaboration and strategic partnerships underpin the country’s leprosy elimination efforts. The Department of Health (DOH), through its Leprosy Control Program, works closely with the World Health Organization (WHO), Regional DOH Centers for Health Development (CHDs), and local government units to coordinate surveillance, treatment, and community engagement. Partnerships with non-governmental organizations (NGOs), including the Philippine Leprosy Mission, Sasakawa Health Foundation, and Novartis Foundation, faith-based groups, academic institutions, Medical Societies (Philippine Dermatology Society), and persons affected by leprosy have strengthened advocacy, case-finding, and psychosocial support. The integration of leprosy within the broader Skin NTDs framework has fostered more efficient use of resources, unified training platforms, and cross-disease surveillance.

Through technical and logistical support from WHO and partners, the country continues to access free multi-drug therapy (MDT) and other essential commodities, monitoring and surveillance to inform evidence-based policy. Sustaining these partnerships remains vital in accelerating progress toward zero leprosy transmission, ensuring that elimination efforts are inclusive, country-owned, community-driven, and sustainable within the Universal Health Care (UHC) system. Conducting research and development to improve diagnostic tools and treatment regimens can help support leprosy elimination efforts [23].

Despite sustained progress, several challenges remain. Persistent stigma, delayed diagnosis, underreporting in remote areas, and disruptions caused by the COVID-19 pandemic continue to affect surveillance and case detection. This paper reviews recent epidemiological trends and examines current program priorities, implementation challenges, and strategic directions for transmission interruption in the Philippines.

## 2. Methods

Quantitative surveillance data were obtained from the Department of Health (DOH) Field Health Services Information System (FHSIS) Annual Reports from 2020–2024. Additional policy and programmatic information was collected from Republic Acts, Administrative Orders, WHO guidelines, national and regional Program Implementation Reviews (2022–2024), and skin neglected tropical disease (NTD) coordination meetings.

To provide a national policy and implementation context, the analysis was anchored on the Philippine Multi-disease Elimination Plan (MDEP) 2024–2030, which integrates leprosy elimination within broader primary healthcare and disease prevention strategies. The alignment between the eight MDEP strategic pillars and the national primary healthcare framework, as seen in Table 1, was used to contextualize program implementation and identify system-level factors influencing elimination efforts.

Qualitative thematic synthesis was guided by the four strategic pillars of the WHO Global Leprosy Strategy 2021–2030: (1) implementing integrated, country-owned zero-leprosy roadmaps; (2) scaling up prevention alongside active case detection; (3) managing leprosy and preventing disability; and (4) combating stigma and ensuring human rights. Information extracted from policy documents, implementation reviews, and stakeholder consultations was categorized according to these pillars. Key implementation enablers and barriers were then identified and synthesized for each pillar (Table 2). The WHO three-phase elimination framework (reduction in disease burden, interruption of transmission, and verification/post-elimination surveillance) provided the broader analytical context for interpreting national epidemiological trends.

Descriptive analyses were conducted to examine trends in prevalence, case detection rates (CDRs), and age-group distribution from 2020–2024. Qualitative findings were synthesized to identify recurring themes related to governance, service delivery, surveillance, financing, stigma reduction, and health system strengthening.

## 3. Results

**Figure 2:**
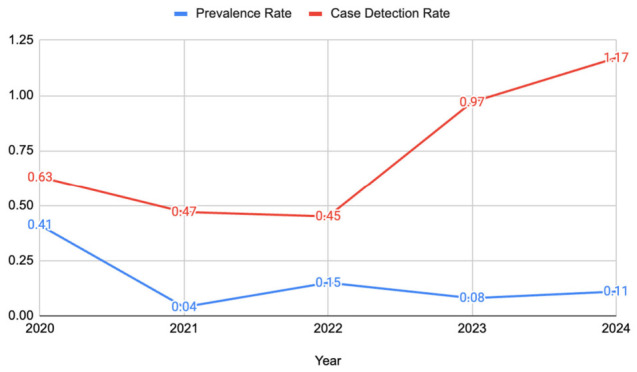
National Prevalence Rate and Case Detection Rate 2020–2024. Note. This figure illustrates the annual trends in the national leprosy prevalence rate and case detection rate from 2020 to 2024, based on the Field Health Services Information System Annual Reports. While the prevalence rate remained below the World Health Organization elimination threshold throughout the study period, the case detection rate increased markedly after 2022, suggesting improved case-finding and surveillance activities. Source: Field Health Services Information System Annual Report [23].

Figure 2 shows the national prevalence and case detection rates for the years 2020 to 2024. We see that the prevalence rate decreased steadily from 0.41 per 10,000 in 2020 to 0.11 in 2024, remaining well below the WHO elimination threshold of 1 per 10,000 population. The sharp decline observed between 2020 and 2021 likely reflected disruptions in surveillance and health service delivery during the COVID-19 pandemic, including reduced access to diagnosis and reporting systems; notably, the 2020 prevalence figure (0.41) represents a statistical outlier and should be interpreted cautiously, as no age/sex disaggregated case data are available for that year, as seen in Table 3. Case detection rates (CDRs) increased from 0.45 per 100,000 population in 2022 to 1.17 in 2024. This rebound likely reflects the resumption of active case-finding, improved surveillance activities, and the integration of leprosy into broader skin NTD programs. Despite this increase, national detection rates remained relatively low overall.

Males consistently represented most of both prevalent and new cases, accounting for 60–66% of annual cases. This pattern is consistent with global epidemiological trends and may reflect delayed health-seeking behavior, occupational exposure, and differences in access to health services.

Most new cases occurred among adults aged 20–59 years, accounting for roughly 70–75% of all reported cases. However, the continued detection of cases among children aged 0–14 years indicates ongoing localized transmission in selected endemic communities. Cases among older adults aged ≥ 60 years (58 total across 2021–2024) may reflect delayed diagnosis or the long incubation period associated with the disease.

## 4. Discussions

The findings demonstrate sustained national control of leprosy in the Philippines while highlighting the need for intensified surveillance in endemic areas. The rebound in case detection following the COVID-19 pandemic likely reflects recovery of public health services rather than a sudden increase in transmission. Similar pandemic-related disruptions to leprosy surveillance and subsequent recovery have been documented in other high-burden countries: global leprosy case detection fell by approximately 37% in 2020 compared to 2019 [25], and comparable rebounds were observed in India, Brazil, and Indonesia following the resumption of active case-finding activities [26,27]. Integration of leprosy activities within broader skin neglected tropical disease (NTD) programs appears to have strengthened case-finding capacity and improved program coordination at both national and subnational levels, consistent with findings from multi-country evaluations of integrated skin NTD approaches in the Western Pacific [28].

The continued detection of pediatric cases (6–7% of new cases aged 0–14 years) remains a significant concern because it suggests ongoing community transmission. This rate is comparable to findings reported from other endemic provinces in Southeast Asia, where child case proportions above 5% are considered a sentinel indicator of active transmission [14,29]. Strengthening contact tracing, early diagnosis, and post-exposure prophylaxis will therefore remain critical to achieving transmission interruption. Single-dose rifampicin (SDR) prophylaxis has demonstrated efficacy in reducing leprosy incidence among contacts in randomized trials [12,30], and its consistent implementation in high-burden areas of the Philippines warrants prioritization. Targeted health education campaigns may also improve health-seeking behavior, particularly among adult males, who consistently account for 60–66% of cases—a disparity documented in global epidemiological reviews [31] and attributed to delayed care-seeking and occupational exposure.

Several operational challenges continue to affect implementation. Persistent stigma remains a major barrier to early consultation and treatment adherence. Geographic isolation, workforce turnover, and uneven local program capacity further contribute to underdiagnosis and delayed case detection in remote provinces. In addition, access to rehabilitation and reconstructive services remains limited outside tertiary centers.

Despite these challenges, the Philippines leprosy program has benefited from strong collaboration between the Department of Health, the World Health Organization, local government units, academic institutions, and nongovernmental organizations. Findings from this study are broadly consistent with programmatic reviews conducted in comparable settings [32] highlighted stigma and geographic access barriers as persistent impediments to leprosy elimination across Asia-Pacific countries, while studies from India and Brazil have documented the impact of decentralization on program quality and underreporting in remote districts [33,34]. The multi-partner model employed in the Philippines—integrating NGOs, academic societies, and WHO technical support—aligns with WHO recommendations for country-owned, community-driven elimination programs. Continued investment in integrated surveillance systems, local capacity building, and sustainable financing will be important for maintaining progress and achieving the national goal of interruption of transmission by 2030.

## 5. Study Limitations

The study’s primary source of data is the annual reports of the Field Health Services Information System of the Epidemiology Bureau (EB) of the Department of Health (DOH), Philippines. The analysis is dependent primarily on secondary data sources largely verified by the Department of Health but may contain some inconsistencies in completeness, accuracy, and reporting quality. The data is largely from public health sources and may not include data from private health facilities, especially those in urban and semi-urban settings. The national representation of the findings must be interpreted cautiously. Concerted efforts should be made through public and private partnerships to promote completeness, quality, and improved decision-making for effective programming.

## 6. Conclusions

The Philippines has maintained leprosy prevalence below the World Health Organization (WHO) elimination threshold while strengthening efforts toward interruption of transmission. Recent increase in case detection likely reflects improved surveillance and recovery of active case-finding activities following pandemic-related disruptions rather than widespread resurgence. Although substantial progress has been achieved, persistent localized transmission, social stigma, and service delivery gaps remain important public health challenges. Sustained investment in surveillance, chemoprophylaxis, early diagnosis, rehabilitation services, and community engagement will be essential to achieving long-term elimination goals.

Moving forward, integrated skin neglected tropical disease (NTD) approaches and stronger local implementation of leprosy programs may help improve efficiency, accessibility, and sustainability. With continued multisectoral collaboration and evidence-based public health strategies, the Philippines is well positioned to advance toward interruption of leprosy transmission and a leprosy-free Philippines by 2030.

## Figures and Tables

**Table 1:** Eight MDEP strategic pillars and the national primary healthcare framework.

	Sectoral Primary Health Care Strategy 2023–2038			Integrated Disease Prevention and Control Through Primary Care Strategic Plan 2023–2028			
MDEP Strategic Pillar	Healthy and Safe Policies & Settings	Accessible, Comprehensive Primary Care for Every Life Stage	Evidence and Data-Informed Response at All Levels	Quality	Access	Self-Sufficient Primary Care	Enabling Mechanism
Surveillance and Information Systems		✓		✓	✓		
Access to Laboratory Services		✓		✓	✓		
Service Delivery		✓		✓	✓		
Safe and Quality Medicines, Vaccines, and Technology		✓		✓	✓		✓
Human Resource and Capacity Building		✓		✓			✓
Environment and Social Determinants of Health	✓						✓
Stewardship and Finance		✓		✓	✓	✓	✓
Research		✓		✓			✓

Integrated approach of the Philippine Multi-diseases Elimination Plan (MDEP), showing the eight strategic pillars underpinning elimination of priority diseases, including leprosy by 2030. Source: Department of Health Philippines, MDEP 2024–2030. Note. Check marks (✓) indicate alignment between the eight strategic pillars of the Philippine Multi-Disease Elimination Plan (MDEP) 2024–2030 and the objectives of the Sectoral Primary Health Care Strategy (2023–2028) and the Integrated Disease Prevention and Control through Primary Care Strategic Plan (2023–2028). The figure illustrates how the MDEP integrates disease elimination efforts, including leprosy, within the Philippine primary healthcare framework. Adapted from the Philippine Multi-Disease Elimination Plan (MDEP) 2024–2030 (Department of Health Philippines, 2024) [9].

**Table 2:** Strategic Pillar-Based Analysis of Enablers and Barriers to Leprosy Elimination in the Philippines.

Strategic Pillar	Enablers	Barriers
1. Implement integrated, country-owned zero leprosy roadmaps	Alignment of national and subnational plans with the WHO Global Leprosy Strategy and NTD Roadmap 2021–2030. Ongoing policy integration through the Administrative Order and Manual of Procedure on Integrated Skin NTDs (Leprosy, Yaws, and Scabies). Strengthened leadership and inter-program collaboration within DOH and Centers for Health Development.	Variability in local program ownership and implementation capacity across regions. Limited domestic funding for surveillance and case management. Weak data feedback between national and subnational levels.
2. Scale up leprosy prevention alongside integrated active case detection	Integration of leprosy screening in skin NTD activities (Kilatis Kutis) and school health programs. Expansion of contact tracing and active surveillance in endemic provinces. Support from WHO and partners for logistics, training, and IEC development.	Gaps in early detection and underreporting due to stigma and limited trained manpower. Inconsistent implementation and integration of chemoprophylaxis (SDR) and post-exposure interventions. Logistical constraints in remote and conflict-affected areas.
3. Manage leprosy and its complications and prevent new disability	Availability of multi-drug therapy (MDT) and supportive commodities through WHO donations. Initiatives on Morbidity Management and Disability Prevention (MMDP) integrated with LF and skin NTDs. Capacity-building of regional and provincial coordinators on case management and treatment.	Insufficient rehabilitation and reconstructive surgery services. Limited follow-up systems for patients’ post-treatment. Unequal access to physiotherapy and self-care support, especially in rural areas.
4. Combat stigma and ensure human rights are respected	Advocacy and health education campaigns led by CHDs and NGOs. Inclusion of leprosy champions and cured persons in awareness events. Communication materials promote inclusion and dignity.	Persistent social stigma and misconceptions about transmission. Limited mainstreaming of human-rights–based approaches in local policies. Lack of sustainable livelihood and reintegration programs for affected persons.

Note. This table summarizes the key enabling factors and implementation barriers across the four strategic pillars of the Philippine leprosy elimination framework. It highlights strengths such as policy alignment, integrated service delivery, and stakeholder collaboration, while identifying persistent challenges including resource limitations, inconsistent program implementation, rehabilitation gaps, and stigma that continue to hinder progress toward national leprosy elimination.

**Table 3:** Leprosy Cases disaggregated by Age Group and Gender.

Year	Population	0–9 Years		10–19 Years		20–59 Years		60 YearsAbove		Total		Total Both Sexes	
		Male	Female	Male	Female	Male	Female	Male	Female	Male	Female	Number	Rate
2020	108,771,978												0.41
2021	110,198,654	67	80	64	42	107	116	20	15	258	253	511	0.46
2022	111,572,254	8	6	11	8	136	87	33	23	188	124	312	0.28
2023	111,638,549	35	17	32	25	128	78	24	24	219	144	363	0.33
2024	112,079,845	4	2	23	11	165	94	35	23	227	130	357	0.32

Note. This table presents the annual distribution of reported leprosy cases by age group and sex from 2020 to 2024. Most cases occurred among adults aged 20–59 years, with males consistently accounting for a greater proportion of reported cases than females. The table also includes annual population estimates, total reported cases, and corresponding national prevalence rates. Source: Field Health Services Information System Annual Report [24].

## Data Availability

Study is based on secondary data derived from programmatic reports and institutional data. No new data was collected. All references are cited. Data from the Department of Health Philippines Field Health Services Information System including Annual Reports (2020–2024) is shown in the DOH webpage: Publications—Department of Health.
